# Clinical and Radiologic Signs of Relapsed Ovarian Germ Cell Tumor: Tissue Is the Issue

**DOI:** 10.1155/2013/984524

**Published:** 2013-10-07

**Authors:** M. Y. V. Homs, H. W. R. Schreuder, G. N. Jonges, P. O. Witteveen

**Affiliations:** ^1^Department of Medical Oncology, University Medical Center, P.O. Box 85500, 3508 GA Utrecht, The Netherlands; ^2^Department of Reproductive Medicine and Gynaecology, University Medical Center, P.O. Box 85500, 3508 GA Utrecht, The Netherlands; ^3^Department of Pathology, University Medical Center, P.O. Box 85500, 3508 GA Utrecht, The Netherlands

## Abstract

Malignant ovarian germ cell tumor is a rare disease, but with current treatment strategies including surgery and platinum based chemotherapy survival is excellent. After treatment, intensive followup is indicated to encounter tumor relapse at an early stage. This case describes a 22-year-old female with a history of common variable immune deficiency (CVID) who underwent a resection of a large ovarian germ cell tumor followed by 4 cycles of cisplatin and etoposide resulting in clinical complete remission. During followup, she developed a mass at the umbilicus and ascites. Initially, the cytology of the ascites was interpreted as tumor positive, suspicious of relapse of the disease, but tumor markers remained negative. However, during laparoscopy it turned out to be a mature teratoma, which can develop after chemotherapy, the so called growing teratoma syndrome. In retrospect, the ascites was false positive. This case shows that current diagnostic tools are not sufficient to distinguish between vital tumor and mature teratoma and can be misleading. Tumor biopsy and/or laparoscopic inspection are therefore indicated.

## 1. Introduction

Malignant ovarian germ cell tumor is a rare disease, which mostly presents in adolescents and young women. With the current management of ovarian germ cell tumors including surgery and platinum based chemotherapy survival is excellent. Five-year survival rate is approaching 100% in early stage disease and at least 75% in advanced stage disease [1, 2]. Therefore, relapse is rare in this population and no standard treatment exists. In addition, during or after chemotherapy mature teratoma can develop, in particular for germ cell tumors with a teratoma compound. This is the so called growing teratoma syndrome or chemotherapeutic retroconversion [3–5]. This case shows that clinical and radiologic signs in combination with cytology can be misleading, suspecting relapsed disease.

## 2. Case Report

**Figure 1:**
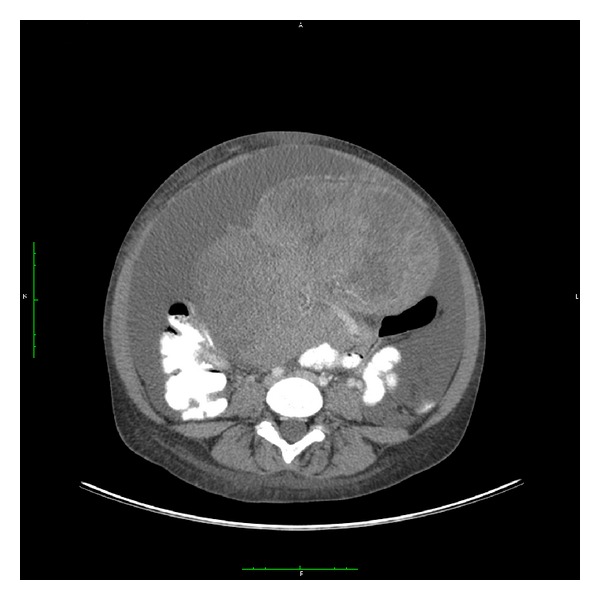
Germ cell tumor.

A 22-year-old female presented with abdominal pain and distension of the abdomen, weight loss, constipation, and fatigue. Her medical history reported a common variable immune deficiency (CVID) with a deficiency of IgG2 subclasses, which was discovered after several pulmonary infections during childhood, resulting in bronchiectasis of the lungs. Since diagnosis she regularly receives immune globulins. An abdominal CT scan identified a mass in the pelvis, likely ovarian origin, and a large amount of ascites (Figure 1). Laboratory results showed an alpha fetoprotein (AFP) of 5300 *μ*g/L (normal range 0.0–9.0), beta human chorionic gonadotropin (B-HCG) of 5800 IU/L (normal range 0.0–3.0), lactic dehydrogenase of 2055 U/L (normal range 0–250), and CA 125 of 136 U/mL (normal range 0–35). She underwent a laparotomy with drainage of 7 liters of ascites, resection of the left ovary with a large tumor that ruptured during surgery, resection of the right fallopian tube, and omentectomy. Inspection of the abdomen showed no signs of residual disease. Pathology showed a mixed germ cell tumor of 25 cm with dysgerminoma, yolk sac tumor, choriocarcinoma, mature teratoma, immature teratoma, and possibly embryonal cell carcinoma. The right fallopian tube contained a cyst and localization of germ cell tumor;the omentectomy showed only reactive changes. In conclusion, she was diagnosed with a mixed germ cell tumor, minimal stage IC. A secondary laparotomy for complete staging was not performed because full macroscopic examination during the first procedure did not show other macroscopic abnormalities, and CT scan performed after surgery showed ascites but no residual tumor. Furthermore, the indication for adjuvant chemotherapy was already there. Because of the preexisting bronchiectasis which has led to reduced lung capacity, bleomycin was contraindicated, and she received 4 cycles of cisplatin and etoposide. At the start of the chemotherapy AFP was 150 ug/L and B-HCG 9.8 IU/L, with still a large amount of ascites. During chemotherapeutic treatment, tumor markers normalized after the third course of chemotherapy and the ascites disappeared without additional paracentesis. Abdominal CT scan 6 weeks after the last chemotherapy showed a minimal amount of free fluid with no signs of residual disease; lymph nodes were all less than 1 cm. Clinical complete remission was concluded and followup started.

**Figure 2:**
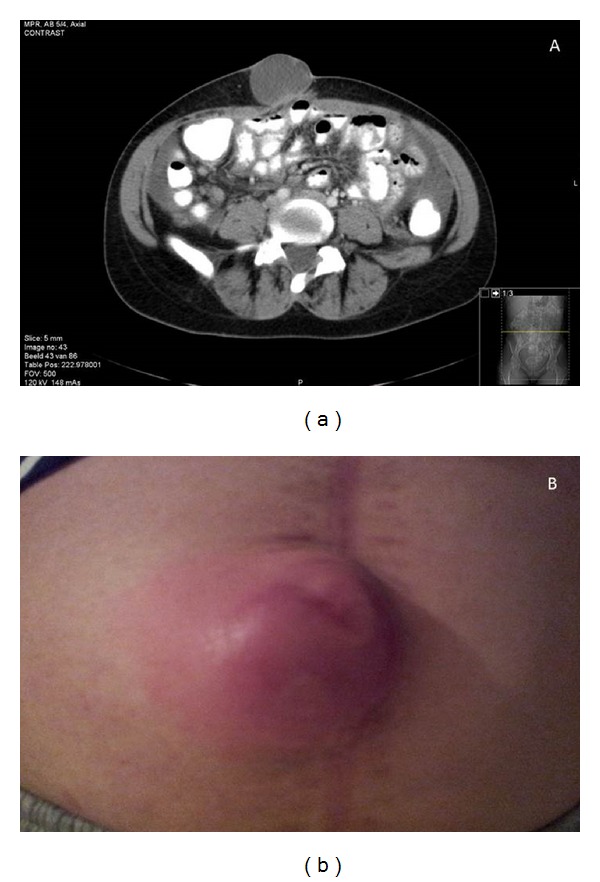
Umbilical swelling.

Only two months later, she developed a swelling at her umbilicus (Figure 2(a)) and regained ascites. She was clinically fit, with no rise in tumor markers. CT scanning confirmed the increased ascites and showed a 4.5 cm lesion at the umbilicus with a similar density to the ascites but with a solid part (Figure 2(b)). FDG-PET scanning showed diffuse moderate uptake in the pelvis next to the uterus and slight lymphadenopathy, mainly mesenterial and inguinal. A biopsy of the lesion at the umbilicus showed fat tissue and connective tissue with reactive changes, no malignancy. Cytology of the ascites was reported to show atypical cells resembling the original germ cell tumor, confirmed by 2 experienced pathologists. The unusual aspects of this case were the negative tumor markers and a clinically fit patient. We considered the diagnosis mature teratoma, but this normally does not present with a large amount of apparently malignant ascites. Due to doubts on the diagnosis we asked our pathologists again for a revision of the cytological material, this time with an immunomarker profile. This showed atypical cells, but immunologic tests on epithelial cell markers (MoC31, Epcam) or germ cell carcinoma (bHCG and aFP) were negative, concluding that this was not enough evidence for relapse of the disease.

**Figure 3:**
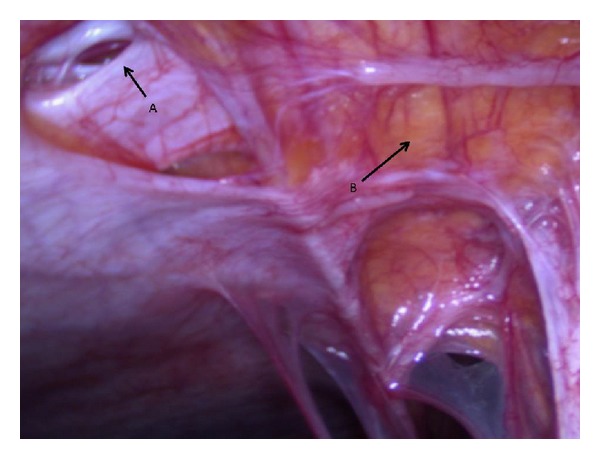
Arrow's: (A) defect in the fascia, communicating with the umbilical swelling; (B) no peritonealization of the abdominal cavity.

**Figure 4:**
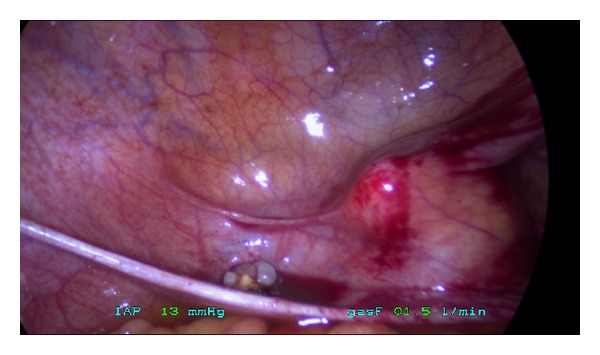
Mature teratoma.

**Figure 5:**
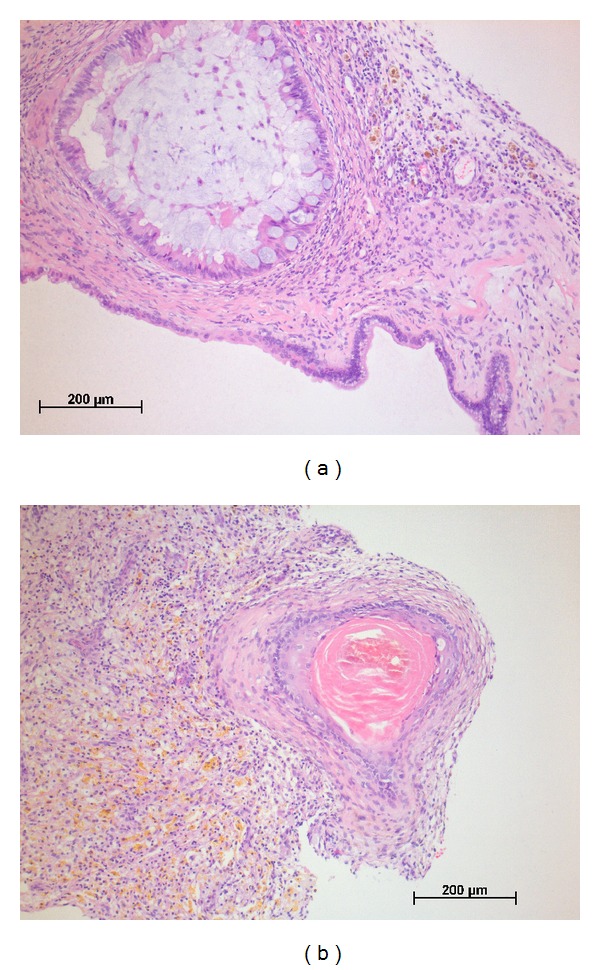
Pathology mature teratoma.

It was decided to perform a diagnostic open laparoscopy. A small fascia defect was detected, with an umbilical hernia (Figure 3(a)). Multiple defects in the peritoneum were seen, which might have caused the ascites (Figure 3(b)). A brown colored mass of 10 mm was removed from the left side of the vesicouterine plica (Figure 4). After complete resection of the brown colored lesion, full inspection of the abdomen showed no further suspected abnormalities, and several biopsies were taken. Histological examination of the brown colored lesion showed a mature teratoma with no signs of immature elements (Figure 5) and no signs of malignancy in the other materials or ascites. Six months after surgery, the patient is clinically fit with no signs of ascites or residual disease and persisting normal tumor markers. 

## 3. Discussion

This case shows that although the patient presented with a swelling at the umbilicus, increased amount of ascites, and initially tumor positive cytology of the ascites, it is still necessary to confirm disease relapse by biopsy, often necessitating laparoscopy. In this case, the clinically fit patient, the history of CVID which might influence radiologic findings and negative tumor markers raised doubts around the diagnosis of relapsed disease.

Growing teratoma syndrome is defined as an increase in tumor size in patients with germ cell tumors during or after chemotherapy, while tumor markers are normal and histology shows only mature teratoma. This phenomenon is also described as “chemotherapeutic retroconversion.” Although this syndrome is well known in males with germ cell tumors, it is rare in females [3–5]. Selective elimination of the malignant cells by chemotherapeutic agents or differentiation of malignant cells into mature teratoma due to the chemotherapy may be the two possible mechanisms. In particular in stage I germ cell tumors with complete excision, this is extremely rare, but might be due to micrometastasis within the peritoneal cavity. Mature teratoma is insensitive for chemotherapy or radiotherapy, and therefore, surgery is indicated. Malignant transformation has been reported in up to 3% of cases [3]. The moderate uptake of the FDG-PET scan did not match the mature teratoma found during laparoscopy, and in our case the PET scan was not able to detect the mature teratoma. From the case series described in the literature, FDG-PET is not sensitive and can either be avid or negative [4, 6]. It is important to recognize the growing teratoma syndrome as it can lead to confusion with progression or relapse of germ cell tumors.

This patient is diagnosed with CVID. Large clinical studies suggest that patients with CVID have a high risk of neoplasms. In particular, the incidence of non-Hodgkin's lymphoma and stomach cancer is increased, but also other solid tumors [7, 8]. No earlier reports on CVID in combination with a germ cell tumor have been described. Due to the CVID with pulmonary infection, she had bronchiectasis with diminished lung function, and therefore, we decided to exclude the bleomycin from the chemotherapeutic treatment. In addition, after every chemotherapeutic course, she received a granulocyte colony-stimulating factor. No infectious problems were encountered during treatment, only a herpes zoster infection 6 weeks after treatment. 

In conclusion, after treatment of germ cell tumors of the ovary, intensive followup is indicated to encounter tumor relapse at an early stage. The development of mature teratoma can be misleading and current diagnostic tools are not sufficient to distinguish between vital tumor and mature teratoma. Tumor biopsy and/or laparoscopic inspection are therefore indicated, in particular in a difficult case as described here. 

## References

[1] Gershenson DM (2007). Management of ovarian germ cell tumors. *Journal of Clinical Oncology*.

[2] Billmire DF, Vinocur C, Rescorla F (2004). Outcome and staging evaluation in malignant germ cell tumors of the ovary in children and adolescents: an intergroup study. *Journal of Pediatric Surgery*.

[3] Hariprasad R, Kumar L, Janga D, Kumar S, Vijayaraghavan M (2008). Growing teratoma syndrome of ovary. *International Journal of Clinical Oncology*.

[4] Byrd K, Stany MP, Herbold NC (2013). Growing teratoma syndrome: brief communication and algorithm for management. *The Australian & New Zealand Journal of Obstetrics & Gynaecology*.

[5] Amsalem H, Nadjari M, Prus D, Hiller N, Benshushan A (2004). Growing teratoma syndrome versus chemotherapeutic retroconversion: case report and review of the literature. *Gynecologic Oncology*.

[6] Kikawa S, Todo Y, Minobe S, Yamashiro K, Kato H, Sakuragi N (2011). Growing teratoma syndrome of the ovary: a case report with FDG -PET findings. *Journal of Obstetrics and Gynaecology Research*.

[7] Park MA, Li JT, Hagan JB, Maddox DE, Abraham RS (2008). Common variable immunodeficiency: a new look at an old disease. *The Lancet*.

[8] Cunningham-Rundles C, Bodian C (1999). Common variable immunodeficiency: clinical and immunological features of 248 patients. *Clinical Immunology*.

